# The gut microbiota in persistent post-operative pain following breast cancer surgery

**DOI:** 10.1038/s41598-024-62397-1

**Published:** 2024-05-30

**Authors:** Khaled Masaud, James M. Collins, Raul Cabrera Rubio, Mark Corrigan, Paul D. Cotter, Niall O’Brien, Ronan Bluett, Clare Keaveney Jimenez, Siobhain M. O’Mahony, George D. Shorten

**Affiliations:** 1 Department of Anaesthesia and Intensive Care Medicine, Cork University Hospital and University College Cork, Cork, Ireland; 2 APC Microbiome Ireland, University College Cork, Cork, Ireland; 3 Department of Anatomy and Neuroscience, University College Cork, Cork, Ireland; 4 Teagasc Food Research Centre, Moorepark, Fermoy, Cork, Ireland; 5 Cork Breast Research Centre, University College Cork, Cork, Ireland; 6 Department of Biotechnology, Institute of Agrochemistry and Food Technology-National Research Council (IATA-CSIC), Valencia, Spain

**Keywords:** Microbiology, Neuroscience, Medical research

## Abstract

Persistent post-surgical pain (PPSP) is defined as pain which continues after a surgical operation in a significant form for at least three months (and is not related to pre-existing painful conditions). PPSP is a common, under-recognised, and important clinical problem which affects millions of patients worldwide. Preventative measures which are currently available include the selection of a minimally invasive surgical technique and an aggressive multimodal perioperative analgesic regimen. More recently, a role for the gut microbiota in pain modulation has become increasingly apparent. This study aims to investigate any relationship between the gut microbiota and PPSP. A prospective observational study of 68 female adult patients undergoing surgery for management of breast cancer was carried out. Stool samples from 45 of these patients were obtained to analyse the composition of the gut microbiota. Measures of pain and state-trait anxiety were also taken to investigate further dimensions in any relationship between the gut microbiota and PPSP. At 12 weeks postoperatively, 21 patients (51.2%) did not have any pain and 20 patients (48.8%) reported feeling pain that persisted at that time. Analysis of the gut microbiota revealed significantly lower alpha diversity (using three measures) in those patients reporting severe pain at the 60 min post-operative and the 12 weeks post-operative timepoints. A cluster of taxa represented by *Bifidobacterium longum*, and *Faecalibacterium prausnitzii* was closely associated with those individuals reporting no pain at 12 weeks postoperatively, while *Megamonas hypermegale*, *Bacteroides pectinophilus*, *Ruminococcus bromii*, and *Roseburia hominis* clustered relatively closely in the group of patients fulfilling the criteria for persistent post-operative pain. We report for the first time specific associations between the gut microbiota composition and the presence or absence of PPSP. This may provide further insights into mechanisms behind the role of the gut microbiota in the development of PPSP and could inform future treatment strategies.

## Introduction

Persistent post-surgical pain (PPSP) is defined as pain which continues after a surgical operation in a significant form for at least three months, and is not related to pre-existing painful conditions^1,2^. PPSP is a common, under-recognised, and important clinical problem which affects millions of patients worldwide^3^. It results from a series of neuroplastic changes sometimes associated with peripheral nerve injury at the time of surgery. While some predisposing factors such as the type of surgery, pre-operative and acute post-operative pain intensity, psychological elements (e.g. pain catastrophising), and genetic factors (e.g. GTP cyclohydrolase 1 haplotype^4^) have been proposed, a reliable predictive tool for this type of pain has yet to be been developed.

Breast cancer is common in Ireland with more than 3000 women and approximately 34 men being diagnosed with the condition each year^5–7^. Surgical management of breast cancer can vary from wide local excision of the tumour to radical mastectomy and a latissimus dorsi reconstruction flap procedure. There is a significant association between the type of surgery these patients undergo and their likelihood of developing PPSP. In particular, axillary node dissection is associated with a greater risk of developing PPSP than less invasive surgeries such as sentinel lymph node biopsy^8^. Patients undergoing breast cancer surgery often experience moderate pain in the days following their surgery, and as many as 80% of those whose surgery includes axillary clearance of lymph nodes experience PPSP^9^. Estimates of the incidence of PPSP after other types of surgery range from 6% (after Caesarean section), to 50–85% (after amputation)^2^.

The gut microbiota, the community of microorganisms within the gastrointestinal tract, has been shown to be an important modulator of a wide range of processes in both health and disease states^10–13^. This community and the microbial metabolites, short-chain fatty acids, and mediators it produces also play vital roles in the bidirectional signalling between the central nervous system (CNS) and the gut via the microbiota-gut-brain axis. This is achieved through several mechanisms including neural, endocrine, and immune system signalling^10,14,15^. While much of the work on the impact of the gut microbiota on the CNS has been defined in animal models, there are now studies in clinical settings indicating that the gut microbiota plays a role in nervous system disorders^16^.

Communication across the gut-brain axis is mediated by microbially-derived molecules including short-chain fatty acids, secondary bile acids, and tryptophan metabolites which interact with the brain through various mechanisms. An example of this is the effect of these molecules on enterochromaffin cells and the regulation of serotonin, an important neurotransmitter in the CNS^17^. In addition, the gut microbiota independently produces a number of neuroactive molecules including dopamine, noradrenaline, and gamma amino butyric acid (GABA) which all play vital roles in CNS function. Furthermore, CNS signalling to the gut microbiota can be directed by stimulating the autonomic nervous system where, through alterations in gut motility and secretions, the gut microbiota may be impacted. Interestingly, a number of studies suggest that the administration of some specific probiotics (live microorganisms that when administered in adequate amounts confer a health benefit on the host^18^) reduces the severity of chemotherapy-induced neuropathy^19,20^. Moreover, the probiotic *Bifidobacterium breve* NCIMB 702258 influences the endocannabinoid system, which has a known involvement in pain perception^21^.

A major modulatory role of the gut microbiota in the sensation of pain has been uncovered in irritable bowel syndrome (IBS) patient cohorts. This specific type of nociception, visceral pain, is a hallmark of IBS and is typified by a diffuse sensation of pain centred around the midline of the body and upper abdomen^22^, and these patients with IBS harbour distinct gut microbiota characteristics^12^. Further, in pre-clinical models manipulation of the gut microbiota reduces visceral hypersensitivity^23–27^, with modest effects being reported in human studies^28^, supporting a role for the gut microbiota in the modulation of pain. Furthermore, some of the pathways and regulators of visceral pain and associated hypersensitivity are also critical in the experience of somatic pain. These include peripheral sensitisation of primary sensory afferents, central sensitisation of spinal ascending neurons, and alteration of descending inhibitory pathways^29^. Moreover, recently a role for the gut microbiota in the regulation of not only visceral pain, but also somatic pain has come to the fore^30^. The gut microbiota has also been found to be important for surgical outcomes^31^ and may be involved in the regulation of chronic pain through the modulation of peripheral and central sensitisation via microbiota-mediated products^30,32^. In both inflammatory pain and neuropathic pain, peripheral sensitisation is an important component of pain regulation which can be triggered by pro-inflammatory mediators such as cytokines or via upregulation of receptors such as toll-like receptors (TLRs)^33^. Manipulation of the gut microbiota in animal models has also been shown to affect both inflammatory and neuropathic pain pathways^19,34^.

We have recently shown in a preliminary study that certain bacteria are associated with persistent pain following upper limb surgery^35^. Although this evidence is limited in extent, it indicates that manipulation of the gut microbiota in the pre-surgical period using probiotics or prebiotics (a substrate (non-digestible fiber) that is selectively utilised by host microorganisms conferring a health benefit^36^), or dietary interventions could reduce the incidence of PPSP.

Hence, given the potential role of the gut microbiota in post-operative pain, we set out to examine this possibility in a different subset of patients. To investigate this, we conducted a prospective observational study of female adult patients undergoing surgery for the management of breast cancer to determine if gut microbiota composition or specific abundances were associated with the incidence and magnitude of PPSP in this patient cohort.

### Primary objective

To determine the association, if any, between pre-defined gut microbiota characteristics and the incidence of PPSP at 12 weeks postoperatively in patients undergoing breast cancer surgery.

### Secondary objectives

To determine the association, if any, between pre-defined gut microbiota characteristics and the severity of acute pain during the first hour after breast cancer surgery.

## Methods

### Study design

#### Recruitment

With institutional ethical approval by the local Ethical Committee of University College Cork (Ref: ECM 4 (e) 04/12/2018) and having obtained written informed consent from each participant, 68 patients (mean age 42.5 years; SD 10.6 years) classified by the American Society of Anaesthesiologists (ASA) physical status classification system as ASA I-III at Cork University Hospital scheduled to undergo breast cancer surgery were included in this study. All patients participated in this study had a body mass index (BMI) less than 35. All methods were performed in accordance with the relevant guidelines and regulations.

#### Inclusion and exclusion criteria

*Inclusion criteria* Female patients of > 17 years old with Irish ethnicity (Irish-born citizen with at least one grandparent born in Ireland) were eligible for inclusion.

*Exclusion criteria* Any contraindication to regional anaesthesia, pain preoperatively of any source requiring analgesic consumption (on more than three occasions) within three months of surgery, history of chronic pain, history of peripheral neuropathy, clinically significant cognitive impairment (as indicated by a MiniMental state score < 24), non-fluency in English, obesity (BMI > 35), antibiotic therapy within the preceding 30 days, probiotic consumption within the preceding 30 days.

#### Faecal sample collection

Participants were provided with collection packs and instructions on how to collect a faecal sample on the day of, or the day before surgery. Samples obtained before the patient came to the hospital were refrigerated prior to transport and participants brought the sample with them to the hospital on the day of their surgery, where they were immediately frozen at – 80 °C until later analysis. No patient was taking antibiotics preoperatively and stool samples were collected prior to the administration of routine prophylactic antibiotic administration.

### Perioperative procedures

Patients completed the Cohen Questionnaire^37^, short-form 6-item Spielberger’s State-Trait Anxiety Inventory (STAI)^38^, and Pain Catastrophising Scale (PCS)^39^ in the week prior to undergoing surgery as well as at 12 weeks postoperatively. An investigator explained the questionnaires and remained available while they were being completed to address any queries.

All patients had standard monitoring applied intraoperatively and underwent general anaesthesia comprising of a standard regimen of propofol 2–3 mg/kg intravenously (iv), fentanyl 1 mcg/kg iv, sevoflurane delivered in an oxygen/air mixture, morphine 0.1–0.15 mg/kg iv, ondansetron 4 mg iv, dexamethasone 8 mg iv, paracetamol 1 g iv, and diclofenac 75 mg iv. Local anaesthetic infiltration of the surgical site was performed by the surgeon (1 mg/kg of bupivacaine 0.5%).

Postoperatively, patients received a continuous wound infusion of bupivacaine 0.2% at 2–8 mL/h (as clinically indicated) for between 12 h and 3 days, regular paracetamol 1 g perorally 6 hourly for four days, regular diclofenac 75mg perorally 12 hourly for four days, and oxycodone 5 mg administered perorally every four hours as required for breakthrough pain. Approximately 60 min postoperatively, each patient completed a short-form McGill Pain Questionnaire (SF-MPQ).

Having received explanation from an investigator, a self-report of pain perception was recorded by each patient in a pain diary from the day of the procedure, weekly until 12 weeks postoperatively.

All patients participated in a phone interview with an investigator at 12 weeks postoperatively during which the patient was asked the following question regarding PPSP: “Do you have pain now which you relate to your surgery (and not arising from other causes)”. In the event that the answer to this question was yes, the patient was asked to describe the severity, intensity, and the characteristics of the pain as detailed in SF-MPQ.

### Microbiota analysis

#### Sample processing, DNA extraction, library preparation and sequencing

DNA was extracted using the QIAmp DNA stool minikit (Qiagen, Crawley, West Sussex, United Kingdom)^40^ following the manufacturer’s instructions with minor modifications and stored at – 80 °C until its use. Prior to shotgun sequencing, a Qubit High Sensitivity DNA assay (BioSciences, Dublin, Ireland) was used to determine the total DNA concentration, and purity was assessed by the 260/280 and 260/230 absorbance ratios using a spectrophotometer NanoDrop ND-1000 (Thermo Fisher Scientific, Wilmington, Delaware). Paired-end sequencing libraries were prepared from the extracted DNA using the Illumina Nextera XTLibrary Preparation Kit (Illumina Inc., San Diego, California) followed by sequencing on the Illumina NextSeq 500, with a NextSeq 500/550 High Output Reagent kit v2 (300 cycles), in accordance with the standard Illumina sequencing protocols.

#### Reads quality filtering

Pre-processing of raw reads by sequence quality and length was performed with PRINSEQ-Lite v0.20.4^41^. A mean quality lower than Q25 in a 10-base pair sliding window was the criteria utilised for trimming low quality reads at the 3ʹ-end. A minimum length of 150 base pairs was ensured for all reads as previously performed^42^. The Illumina sequences were screened against the Human reference genome (Homo sapiens UCSC) downloaded from Illumina iGenomes^43^ to remove host reads using Bowtie2 (version 2.2^44^) using the default values, in order to identify and remove host DNA sequences and reads derived from possible human DNA contamination. The unmapped reads were then used for the downstream analysis in accordance with existing literature^45^.

Read duplicates were removed using the Picard MarkDuplicates tool^46^ to create FASTQ files with unique reads only. Afterwards, reads were subjected to a further quality filtering step. In brief, sequences were trimmed for low quality score using a modified version of the script trimBWAstyle.pl that works directly from BAM files (TrimBWAstyle.usingBam.pl, 2010; https://github.com/genome/genome/blob/master/lib/perl/Genome/Site/TGI/Hmp/HmpSraProcess/trimBWAstyle.usingBam.pl). The script was used to trim off bases with a quality value of three or lower. This threshold was chosen to delete all the bases with an uncertain quality as defined by Illumina’s EAMMS (End Anchored Max Scoring Segments) filter. Additionally, reads trimmed to less than 200bp were also removed as previously performed^42^.

The analysis of the microbial composition was carried out using the MetaPhlAn2 species classifier^47^. Taxa with a relative abundance of < 0.01% were categorised as "Other" for each classifier. Subsequently, the functions were assigned with HUMAnN2 tool^48^, based on ChocoPhlan and UniRef databases^49^. The HUMAnN2 gene abundance table was regrouped by a mapping of KO terms for all categories of bacterial metabolism and dividing the functional table into two files (one stratified and one non-stratified). Metagenome assembly was performed using MEGAHIT^50^.

### StrainPhlAn

StrainPhlAn^51^ is a tool for identifying the specific strain of a given species within a metagenome. This tool is designed to track strains across large collections of samples and takes the raw metagenomic reads in FASTQ format as input. After mapping the reads against the set of species-specific markers (> 200 per species), StrainPhlAn reconstructs the sample specific marker loci using a variant calling approach and outputs the sequences of each sample-specific marker in FASTA format. Sequences are extracted from the raw reads using a reference-free majority rule that filters out noisy regions^52^.

The resulting sequences were then concatenated and aligned by StrainPhlAn with Muscle version 3.8^53^. In this work, we applied StrainPhlAn in 4 species. The reconstructed markers were used to build the phylogenetic tree. StrainPhlAn version 1.0 was used with default parameters, using the mpa_v20 m200 markers database of MetaPhlAn2^54^. The mapping against the markers was performed with BowTie2, version 2.2.6, with the parameters implemented in the StrainPhlAn pipeline^44,52^.

### Statistical analysis

The results from the MetaPhlAn2 output were used for taxonomic profiling. Functional assignment was performed at the KO gene level. Statistical analyses were carried out with R (R version 3.6). To perform alpha diversity analyses (Richness, Shannon Index, and Simpson Index), as well as the multidimensional scaling analysis (MDS) based on Bray–Curtis, the statistical package “vegan” version 2.3.0^55^ was used. The Adonis function in “vegan” was used for PERMANOVA (permutational analysis of variance) analysis, and betadisper function for MDS figures. To compare alpha diversity metrics among groups, ANOVA (aov) test was performed and was adjusted with the Benjamini–Hochberg method as previously performed^56^. In addition, linear models were constructed to evaluate the influence of different environmental variables on these outcomes. Linear discriminant analysis effect size (LEfSe) was performed in order to discover specific bacterial biomarkers associated with pain. Bacterial interrelations were evaluated by calculating Spearman’s correlations between dominant species in raw data and contigs. Finally, Random Forest (RF) was used for building case–control classification models including all microbiological variables. Benjamini–Hochberg method was used for p-values corrections in case of multiple testing. DESeq2 procedure^57^ was used to calculate differentially abundant functional results. Functional results were tested with a false discovery rate (FDR) or the expected proportion of false positive findings). Spearman’s correlation was calculated between rlog normalised functional cpm. The phylogenetic trees of *Bifidobacterium alolescentis*, *Bifidobacterium longum*, *Escherichia coli*, and *Faecalibacterium prausnitzii* were created in GraPhlAn^58^, using the StrainPhlAn^51^ output, which used Metaphlan2 taxonomic assignment^51^.

The threshold of statistical significance was established as p < 0.05 (*). Other notable significance values include p < 0.01 (**) and p < 0.001 (***), p < 0.0001 (****), based on the corrected p-values.

## Clinical results

**Figure 1:**
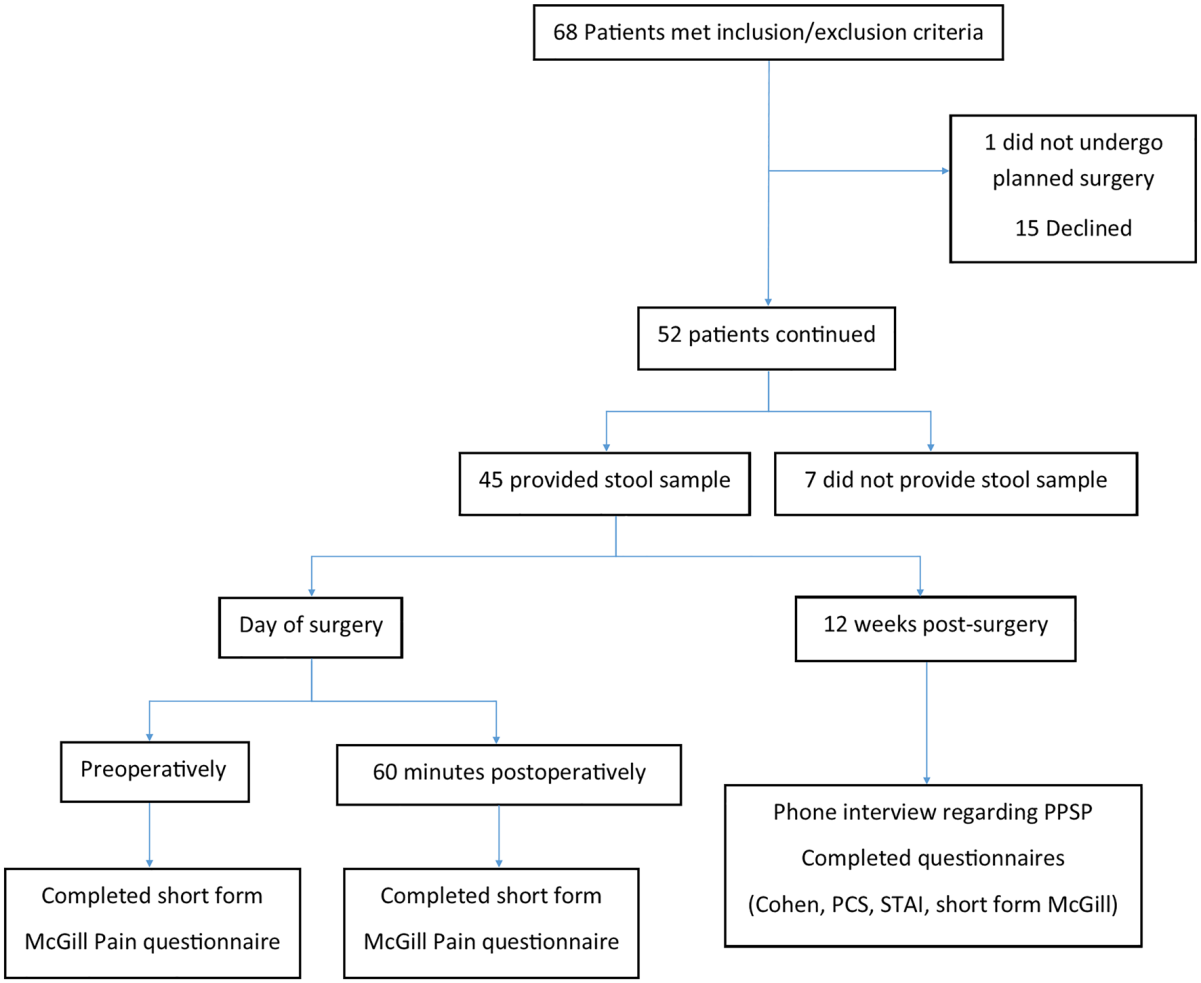
Flowchart of participants recruited, and data collected during the study. *PCS* pain catastrophising score, *PPSP* persistent post-surgical pain, *STAI* state-trait anxiety inventory.

Data were collected from 68 patient (mean age 42.5 years; SD 10.6 years). Most participants [45 patients (66.17%)] provided a stool sample for microbiome analysis and completed the study. Fifteen patients (22.05%) declined to proceed after providing consent, and one patient (1.47%) consented but did not undergo the planned surgery, while seven patients (10.29%) were willing to participate but were not able to provide a stool sample, and so were excluded in the final analysis (Fig. 1).

**Figure 2:**
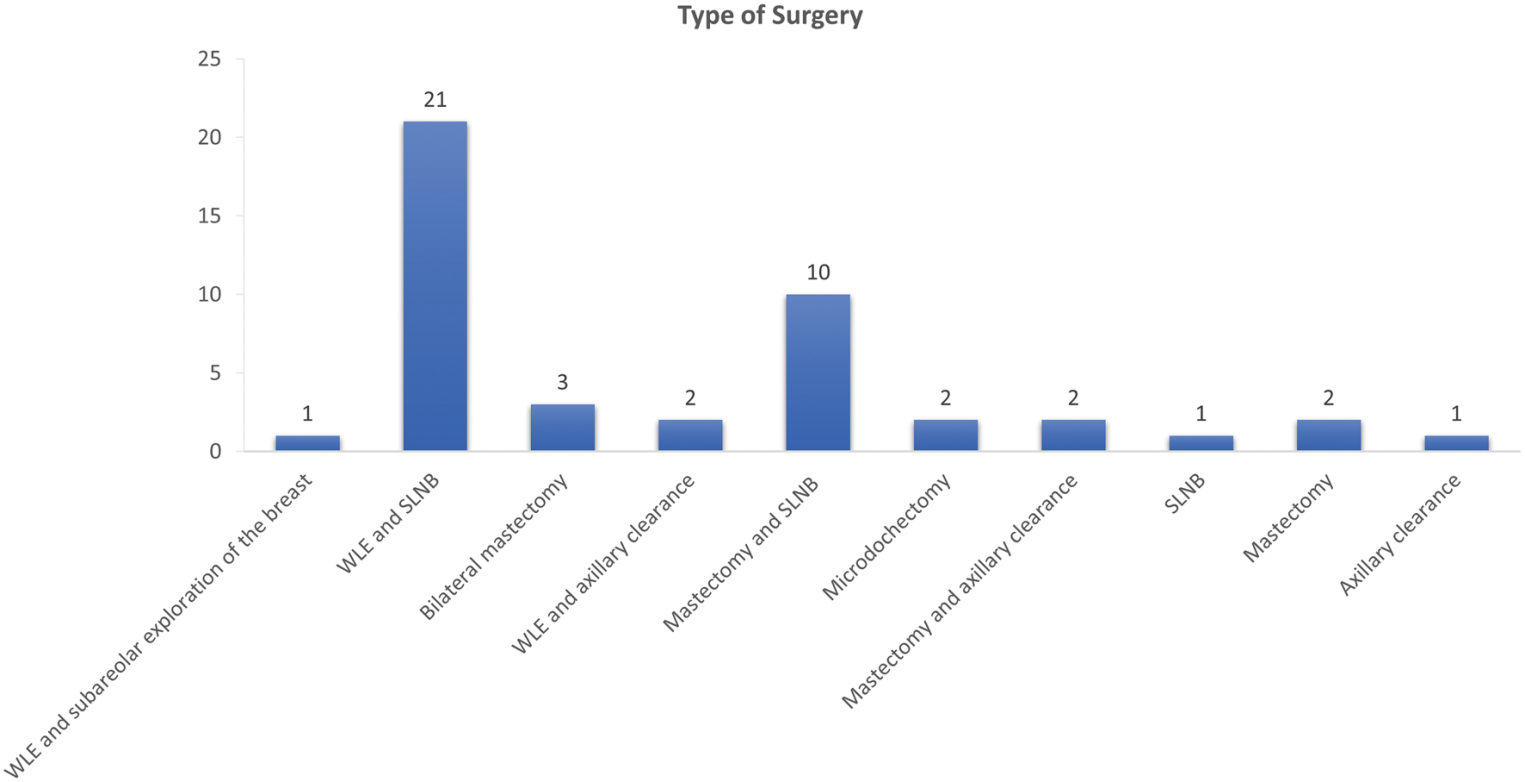
Breakdown of the type of surgery the participants underwent. WLE; wide local excision, SLNB; sentinel lymph node biopsy.

Twenty-one (46.6%) patients underwent wide local excision (WLE) and sentinel lymph node biopsy (SLNB), 10 (22.2%) underwent mastectomy and SLNB, and two (4.4%) underwent unilateral mastectomy (Fig. 2).

### McGill scores over time

Preoperatively, 60 min postoperatively, and 12 weeks postoperatively, patients were asked to complete the SF-MPQ. On the main component of the SF-MPQ questionnaire, total scores were calculated comprising the sum of sensory and affective intensity rank values and categorised as follows: 0–no pain, 1–3 mild pain, > 3–5 moderate pain, and > 5 severe pain. Amongst the 45 patients who provided a stool sample, 1 patient (2.2%) preoperatively, 2 patients (4.4%) at 60 min postoperatively, and 4 patients (8.8%) at 12 weeks postoperatively did not provide a response to the SF-MPQ questionnaires. Of those who responded, most patients reported no pain preoperatively, increased pain intensity at 60 min after surgery, and pain of lesser intensity at 12 weeks after surgery (Table 1). Pre-operation, most patients (68.8%) felt no pain in the three domains of SF-MPQ. 60 min postoperatively the largest group (33%) reported moderate pain at rest C1. At 12 weeks postoperatively, 21 patients (51.2%) did not have any pain and 20 patients (48.8%) reported feeling pain that persisted at 12 weeks post-surgery.

**Table 1:** Pain Rating Index pre- and postoperatively.

	PRI pre-operatively	PRI 60 min postoperatively	PRI at 12 weeks postoperatively
Mean	0.34	1.41	0.68
SD	0.68	0.85	0.84

PRI is Pain Rating Index based on self-report of pain experienced using the short-form McGill pain questionnaire (SF-MPQ) calculated as the sum of sensory and affective intensity rank values.

### Psychological states over time

Patients were asked to complete questionnaires aimed at gauging their psychological state pre- and postoperatively. One patient (2.2%) preoperatively, and one patient (2.2%) postoperatively did not provide responses to the questionnaires. The distribution of responses shows that state anxiety, trait anxiety, and catastrophising remained largely unchanged between prior to, and 12 weeks after surgery. Forty percent of patients scored 40 and above in pre-operative state anxiety, and 24.4% scored 40 and above in pre-operative trait anxiety, compared to 28.8% of patients who scored 40 and above on the state anxiety questionnaire with 24.4% scoring 40 and above in trait anxiety at 12 weeks after surgery. Most patients reported low catastrophising both before (98%) and after (95%) surgery, unlike stress which increased markedly postoperatively (Table 2).

**Table 2:** State and trait anxiety, stress, and catastrophising scores of patients pre- and postoperatively.

	Pre-operative state anxiety	Post-operative state anxiety	Pre-operative trait anxiety	Post-operative trait anxiety	Pre-operative Cohen	Post-operative Cohen	Pre-operative PCS	Post-operative PCS
Mean	38	35.2	33.7	36.1	12.6	13.7	8.5	9.6
SD	13	12.1	9.1	11.6	6.4	7.2	9.1	11.2

Pre- and post-operative state anxiety refer to values elicited for the state component of the Spielberger State-Trait Anxiety Inventory during the week before and 12 weeks after surgery respectively; pre-and post-operative trait anxiety refer to values elicited for the trait component of the Spielberger State-Trait Anxiety Inventory during the week before and 12 weeks after surgery respectively. Pre-and post-operative Cohen refer to values elicited for stress using the Cohen Questionnaire during the week before and 12 weeks after surgery respectively. Pre- and post-operative PCS refer to values elicited for catastrophising using the Pain Catastrophising Scale during the week before and 12 weeks after surgery respectively. No significant pre- versus post-operative changes were observed for any of these parameters.

### Taxonomic analysis from metagenomic raw data

#### Alpha diversity and richness

The Richness Index showed significantly lower richness for severe present pain intensity (PPI) versus mild PPI at the 60 min post-operative timepoint, with somewhat lower significance observed at the 12 weeks timepoint for severe versus mild PPI (Table 3). The Shannon index demonstrated significantly lower gut microbiota alpha diversity for those patients reporting severe PPI versus those who reported mild PPI at 60 min and 12 weeks postoperatively (Table 3). The Simpson Index also demonstrated lower gut microbiota alpha diversity for patients reporting severe versus mild PPI (Table 3). No significant difference in gut microbiota alpha diversity was identified in those patients who did not meet the criteria for PPSP (Table 3).

**Table 3:** Alpha diversity was noted to be increased in those patients reporting mild pain which it was decreased in those reporting severe pain at both the 60 min post-surgery and 12 weeks following surgery time points.

McGill pain questionnaire score	^#^Of patients	Measure of diversity (Mean ± SEM)		
		Richness	Shannon Index	Simpson Index
60 min postoperatively C1				
No pain	11	0.612 (± 0.205)	1.757 (± 0.592)	0.729 (± 0.239)
Mild pain	7	0.689** (± 0.057)	2.016** (± 0.172)	0.827** (± 0.034)
Moderate pain	21	0.622 (± 0.107)	1.848 (± 0.368)	0.752 (± 0.126)
Severe pain	4	0.335** (± 0.277)	1.032** (± 0.862)	0.427** (± 0.378)
12 weeks postoperatively C2^#^				
No pain	21	0.612 (± 0.158)	1.793 (± 0.479)	0.74 (± 0.183)
Mild pain	6	0.677* (± 0.059)	2.071** (± 0.193)	0.831* (± 0.025)
Moderate pain	10	0.581 (± 0.198)	1.697 (± 0.583)	0.684 (± 0.234)
Severe pain	1	0.606* (± 0.001)	1.845** (± 0.001)	0.801* (± 0.001)
Presence of PPSP				
Yes	18	0.591 (± 0.204)	1.842 (± 0.443)	0.705 (± 0.243)
No	23	0.634 (± 0.09)	1.787 (± 0.48)	0.77 (± 0.097)

C1 refers to the verbal rating scale at rest component of the SF-MPQ, C2 refers to the verbal rating scale with movement component of the SF-MPQ. Asterisks indicate a significantly lower value in the severe pain versus mild group (*p < 0.05 and **p < 0.01). PPSP; Persistent post-surgical pain. ^#^Of the 41 patients who provided responses to the SF-MPQ at 12 weeks postoperatively, 3 did not include responses to the C2 section.

#### Beta diversity

**Figure 3:**
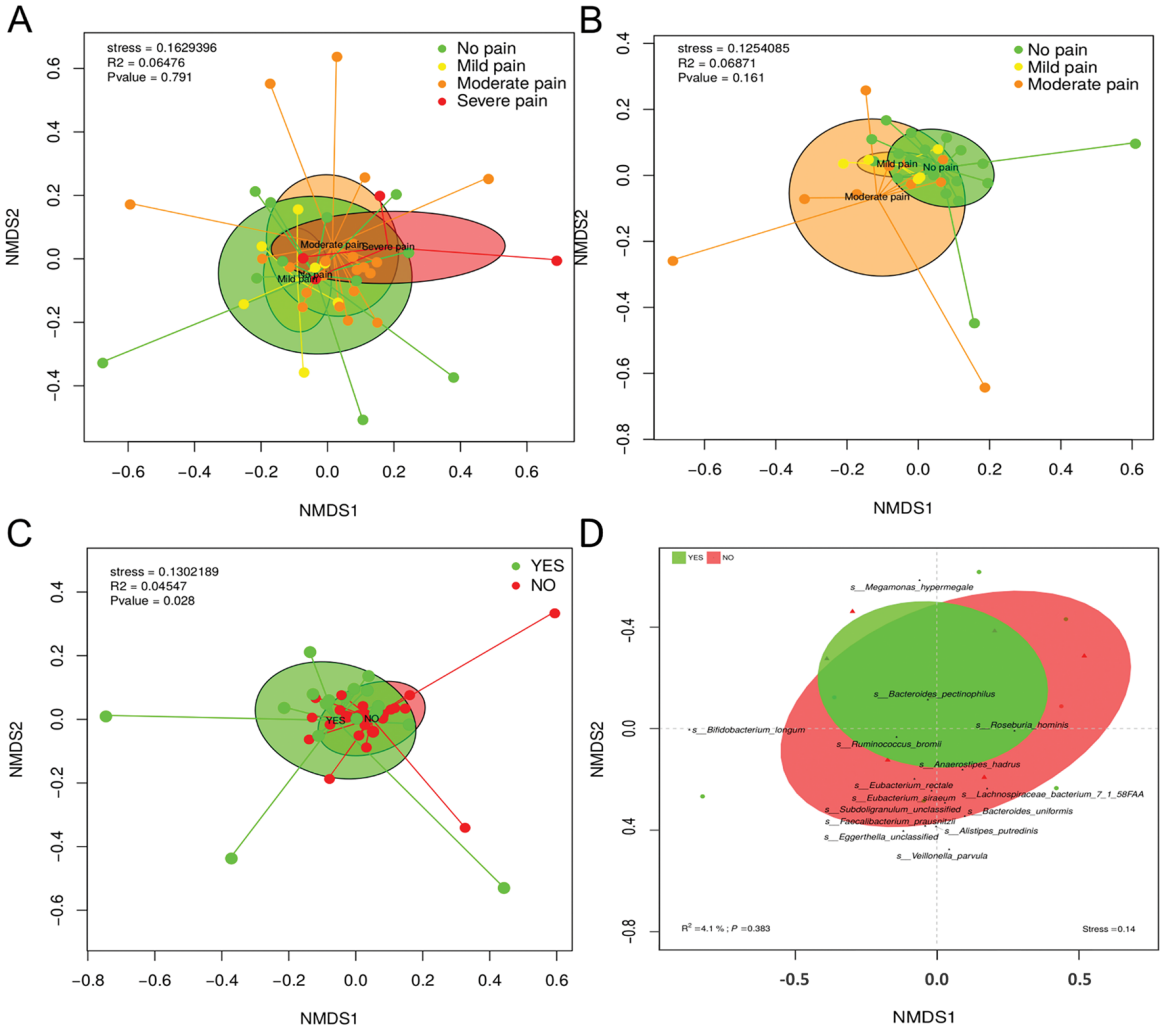
Beta diversity of the gut microbiota (**A**) 60 min postoperatively (**B**) 12-weeks postoperatively (**C**) associated with the presence or absence of persistent post-surgical pain (**D**) A bi-plot of the bacterial species associated with those individuals fulfilling the criteria for the presence of PPSP (yes) or not (no).

The comparison of the taxonomic profile of the samples by using the Bray–Curtis dissimilarity index revealed the combined influence of sample type and PPSP (adonis2, p < 0.028) in the clustering and ordination of samples at family level (Fig. 3). However, no significant differences were observed at the family level in samples from participants at 60 min and 12 weeks postoperatively (Fig. 3A,B, respectively), but a non-significantly different distribution was observed according to the type of pain. The gut mi﻿crobiota composition at the species level according to pain status using a Bray–Curtis dissimilarity index was not significant, but the relationship between the presence or absence of post-surgical pain and changes in beta-diversity was assessed (Fig. 3D). A cluster of taxa *Bifidobacterium longum*, *Faecalibacterium prausnitzii*, two species of *Eubacterium*, *Subdoligranolum*, *Veillonella parvula* and other species formed part of the no PPSP group (p = 0.05). Finally, *Megamonas hypermegale*, *Bacteroides pectinophilus*, *Ruminococcus bromii* and *Roseburia hominis* clustered relatively closely in the PPSP group (p = 0.05) (Fig. 3D).

#### Taxonomy at genus level

At the genus level, distribution of abundance varied significantly across the patient groups who did and did not meet the criteria for PPSP. For instance, the mean abundance of Bifidobacteria was significantly greater in patients with PPSP (at 12 weeks postoperatively) while the mean abundance of *Bacteroides* was significantly greater in those who did not have PPSP (data not shown) and *Eubacterium*, *Bacteroides*, *Bifidobacterium*, *Ruminococcus*, *Escherichia*, *Prevotella,* and *Streptococcus* were the most abundant genera in cases with PPSP (data not shown). In contrast, the most abundant genera in cases without PPSP were *Bacteroidales*, *Catenibacterium*, *Sacharibacteria*, *Butyrivibrio,* and *Lachnospiraceae* (data not shown)**.**

**Figure 4:**
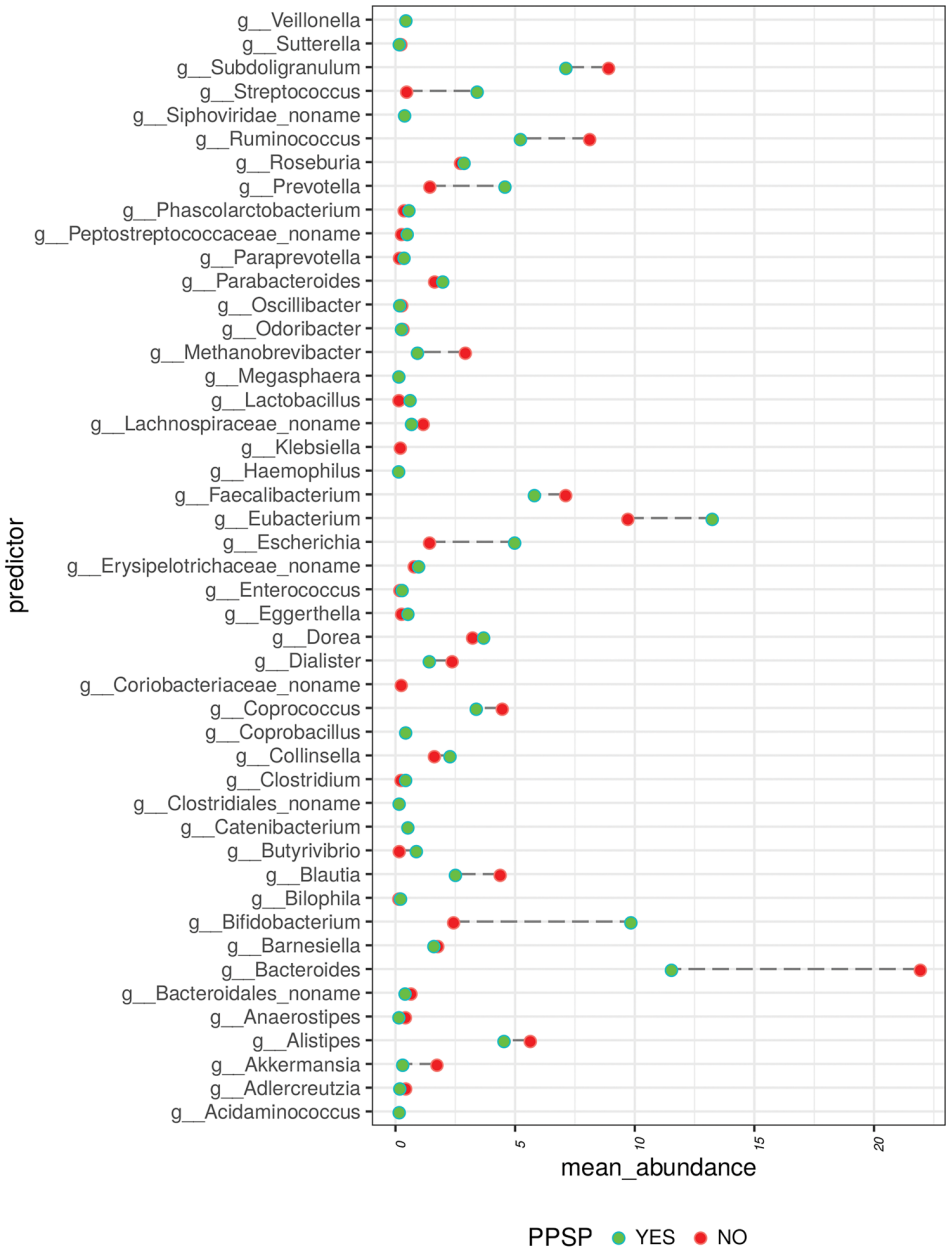
Representation of differential abundance of genera between PPSP (yes) and no pain (no) groups.

The greatest differences between both groups are observed between genera such as *Bacteroidales, Bifidobacterium*, *Blautia, Butyrivibrio, Coprococcus, Escherichia, Eubacterium, Faecalibacterium, Methanobrevibacter, Prevotella, Ruminococcus, Streptococcus, and Subdoligranulum* (Fig. 4).

#### LEfSe

**Figure 5:**
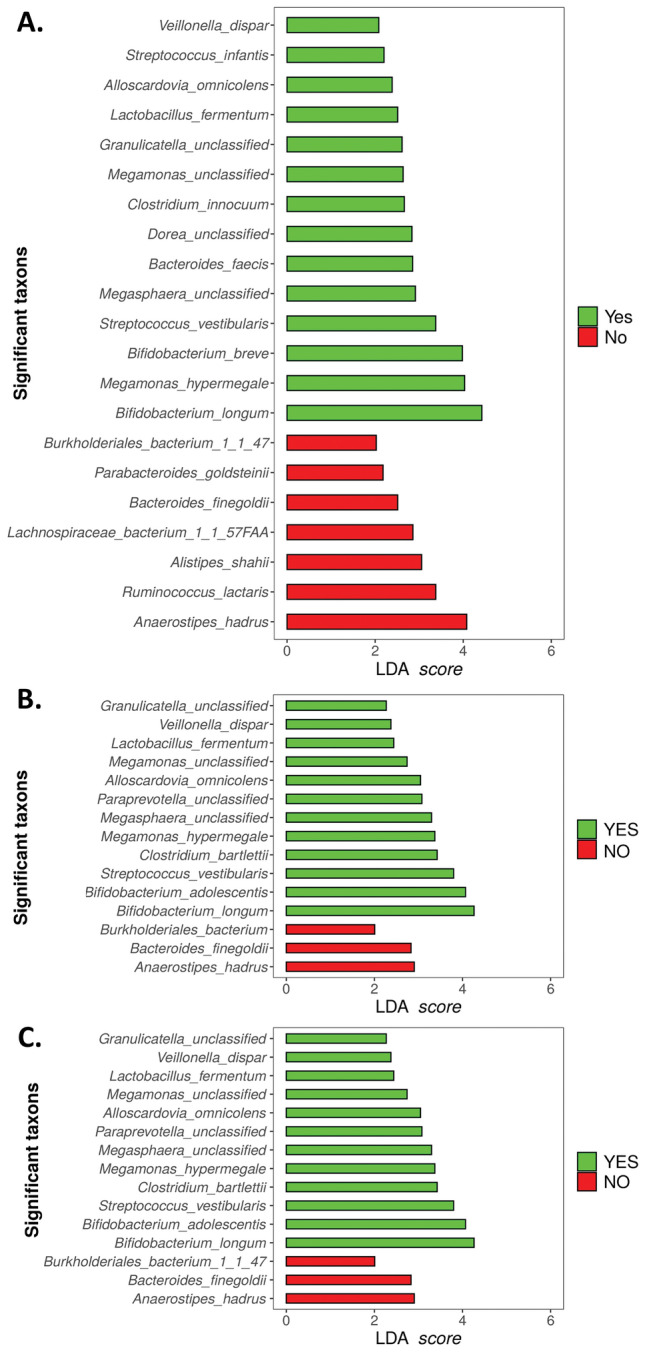
Representation of species associated with pain post-surgical procedure (green), and species associated with the absence of this pain (red) (**A**) At 12-weeks postoperatively according to pain status and following the Mc Gill questionnaire (**B**) 60 min and (**C**) 12 weeks postoperatively.

Analysis of the differential distribution of species according to pain status was assessed with LEfSe. Among the most abundant species (relative abundance > 0.01%), LEfSe identified *Anaerostipes hadrus*, *Ruminococcus lactaris,* and *Alistipes shahii* as overrepresented in the group without PPSP, while *Bifidobacterium longum*, *Megamonas hypermegale*, *Bifidobacterium breve*, and *Streptococcus vestibularis* were overrepresented in the PPSP group at 12 weeks postoperatively (Fig. 5A).

LEfSe also corroborated the association of bacterial species from the SF-MPQ 60 min and 12 weeks postoperatively, where species including *Anaerostipes hadrus*, *Bacteroides finegoldii,* and *Burkholderiales bacterium* were highly represented in the no pain group at 60 min and 12 weeks postoperatively (Fig. 5B,C). On the contrary, the species found to be overrepresented in the pain group at 60 min and 12 weeks postoperatively were *Bifidobacterium longum*, *Bifidobacterium adolescentis*, *Streptococcus vestibularis*, *Clostridium bartlettii*, *Megamonas hupermegale*, *Megasphaera unclassified*, *paraprevotella unclassified*, *Alloscardovia omnicolens*, *Megamonas unclassified*, *Lactobacillus fermentum*, *Veillonella dispar,* and *Granullicatella unclassified* (Fig. 5B,C).

### Associations between gut microbiota and PPSP

**Figure 6:**
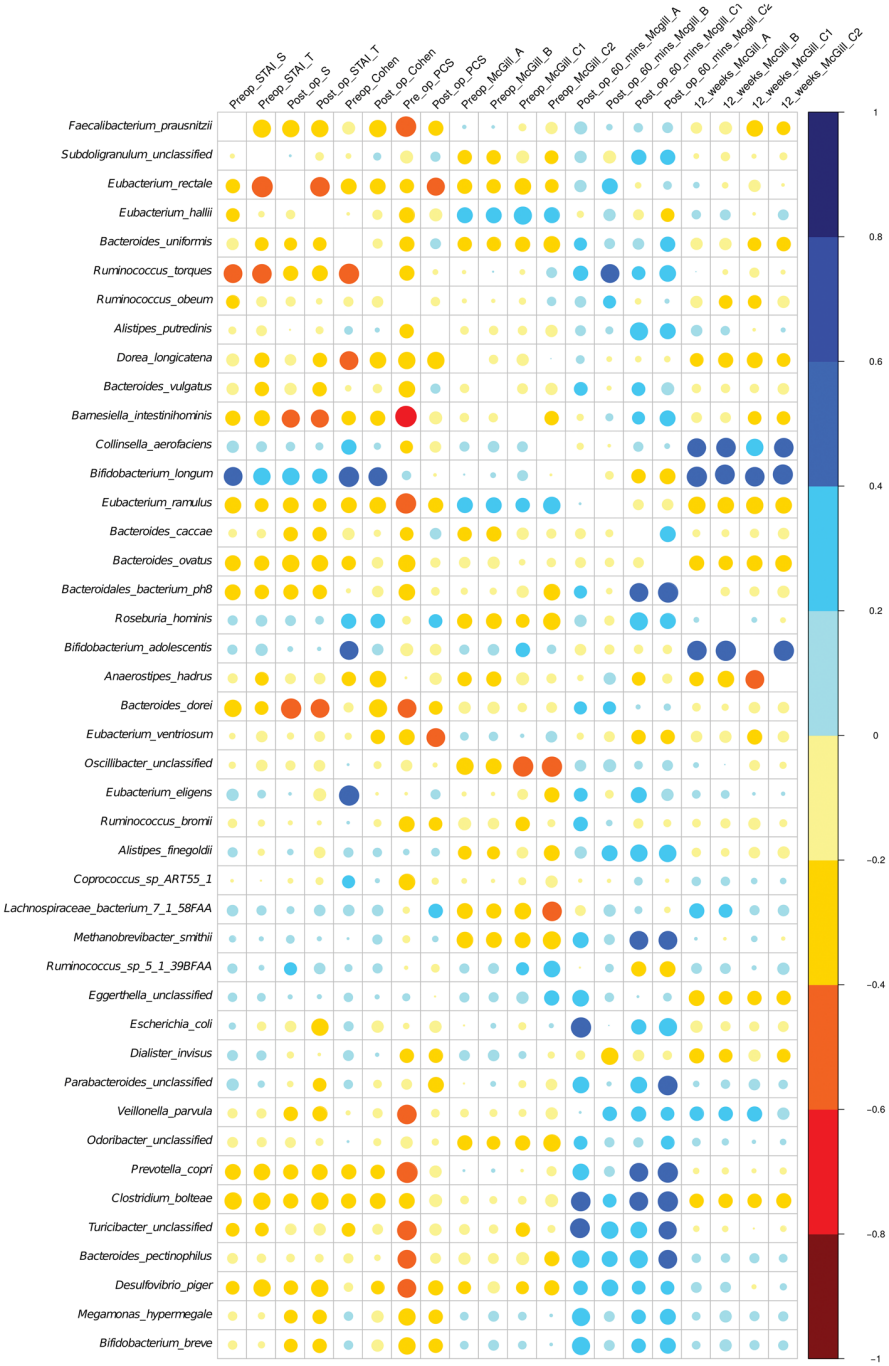
Heat map plot representing associations between gut microbiota species and pain post-surgical procedure.

The heat map plot (Fig. 6) represents a real-valued similarity matrix showing specific associations between microbes and all pain indices examined in this study for each participant. Several positive correlations were found for all the indices with the species *Bifidobacterium longum*, *Bifidobacterium adolescentis*, *Megamonas hypermegale*, *Bifidobacterium adolescentis,* and *Collinsinella aerofaciens*. Although all of these correlations are interesting and striking, none of these observations met the threshold of statistical significance.

Of note, *Bifidobacterium longum* abundance was highly significantly associated with the magnitude of pain experienced at 12 weeks as expressed using SF-MPQ A, B, C1 (PPI) and C2 (Verbal Rating Scale for average pain intensity) (Fig. 6).

### Differential expression of genes associated with the presence or absence of PPSP

**Figure 7:**
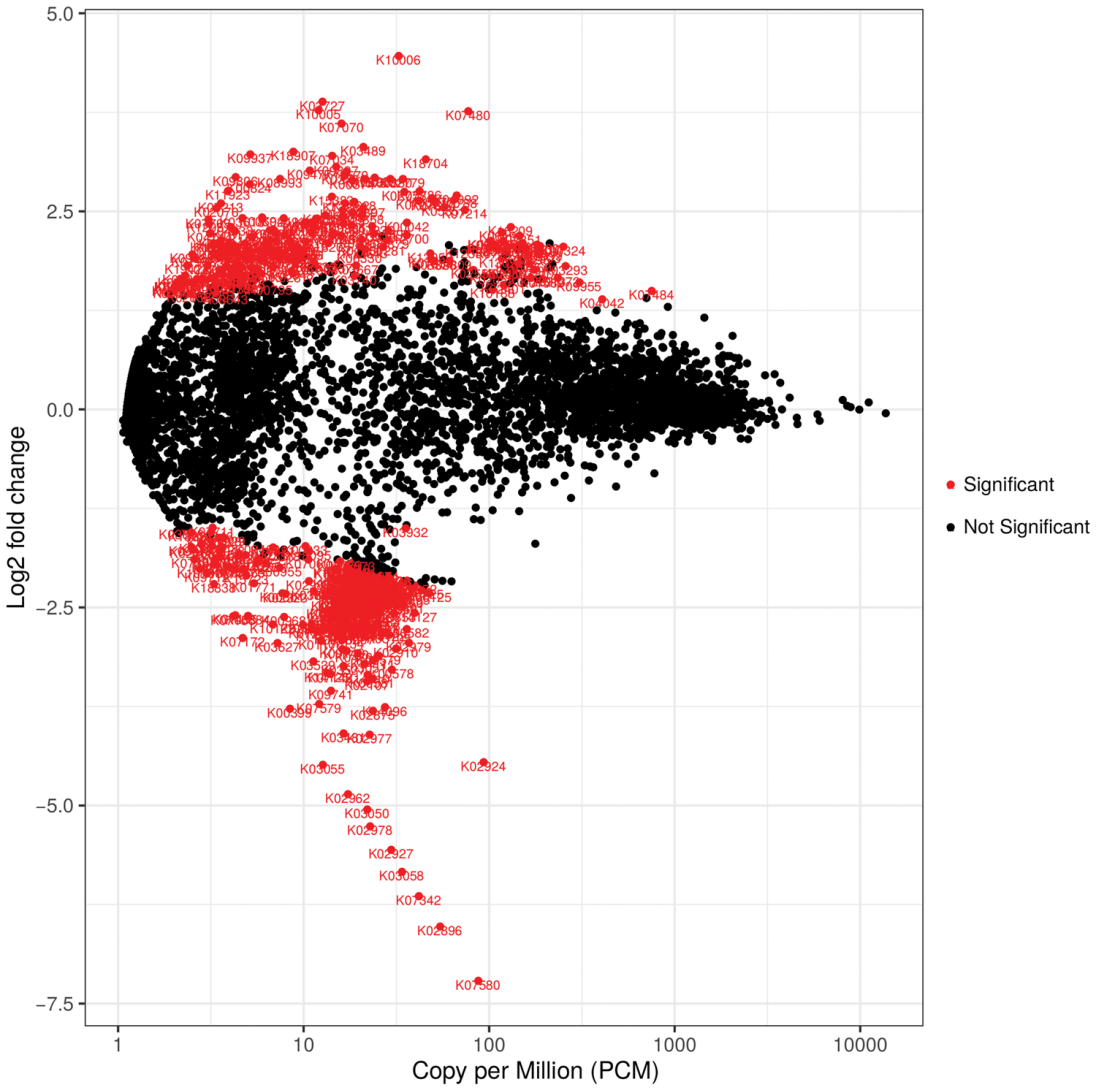
Volcano plot displaying significantly differentially expressed genes (red) relevant to functional pathways between pain and no pain groups.

The volcano plot shows surface p values after FDR correction versus log2 fold change (LFC) of functional pathway abundance. Despite an even distribution of pathway superclasses, several were significantly different when the functions of the KO/GO databases between pain and no pain in the no post-surgical pain group were assessed. The positive y axis (y > 0) shows the genes that were upregulated, and the negative y axis (y < 0) shows the genes that were downregulated. The significantly differentially expressed genes are shown in red (Fig. 7). Further analysis assessing specific species was also carried out (Fig. S1).

### Taxonomic analysis from metagenomics from MAGs

**Figure 8:**
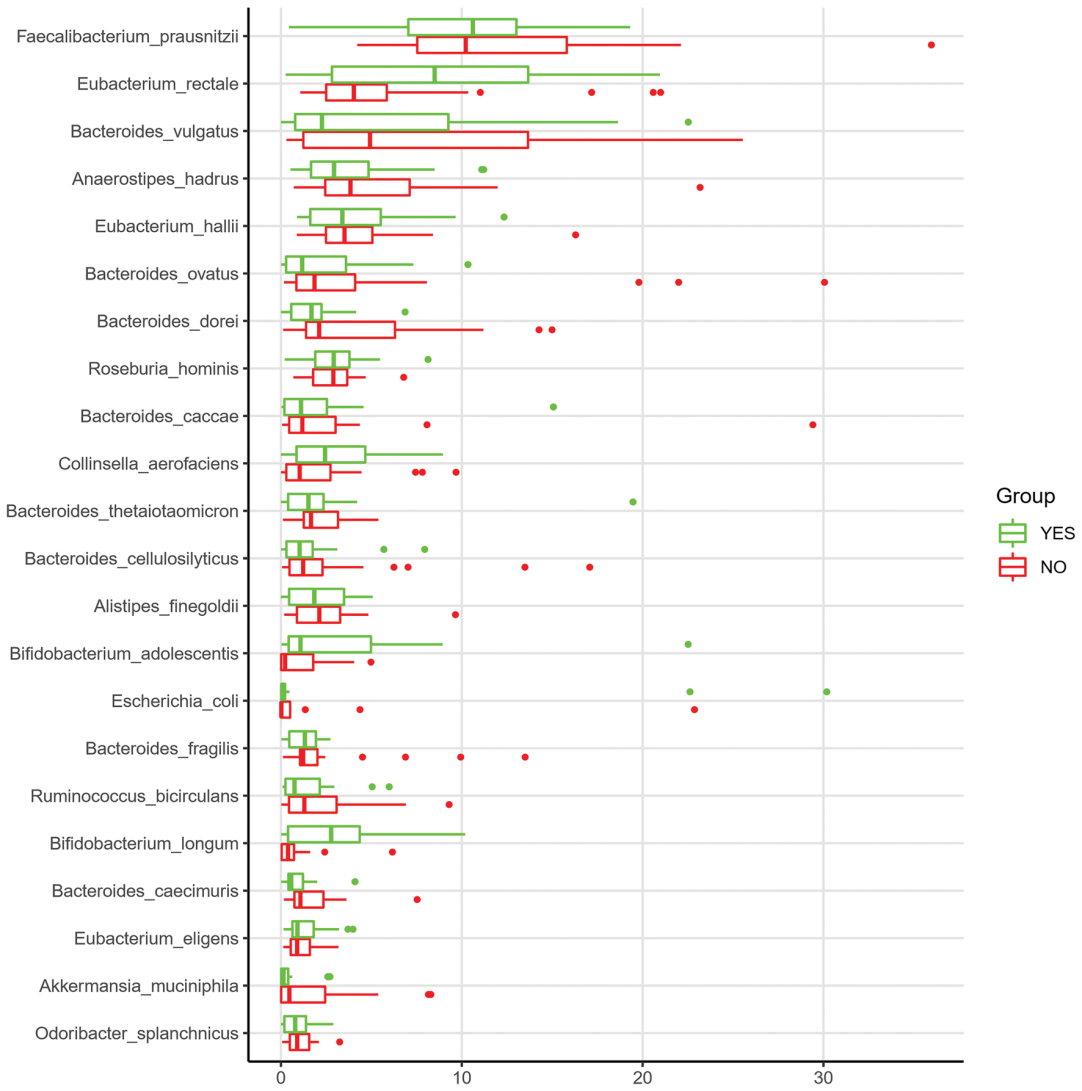
Taxonomic analysis from metagenomics from MAGs associated with the presence (yes) or absence (no) of PPSP.

Contigs were predicted to belong to 253 different bacterial species with a concentration greater than 0.001%, with *Faecalibacterium prausnitzii*, *Eubacterium rectale*, *Bacteroides vulgatus*, *Anaerostipes hadrus*, *Eubacterium halli*, *Bacteroides ovatus*, *Bacteroides dorei*, *Roseburia hominis*, *Bacteroides caccae*, *Collinsella aerofaciens*, *Bifidobacterium adolescentis*, *Bifidobacterium longum*, *Akkermansia muciniphila* and other species shown in Fig. 8. These Species account for 70.56% of the total of Species abundance in the contigs. We further depicted the difference in abundance between those individuals suffering from PPSP or not. Figure 8 shows the differences between these groups with respect to the percentage of assembled contigs greater than 500 bp between both groups.

### Contigs alpha- and beta-diversity analysis

The alpha-diversity indices were calculated for the SF-MPQ PPI scores after 60 min and 12 weeks postoperatively, and also for the presence/absence of PPSP. Neither index was significant (data not shown).

**Figure 9:**
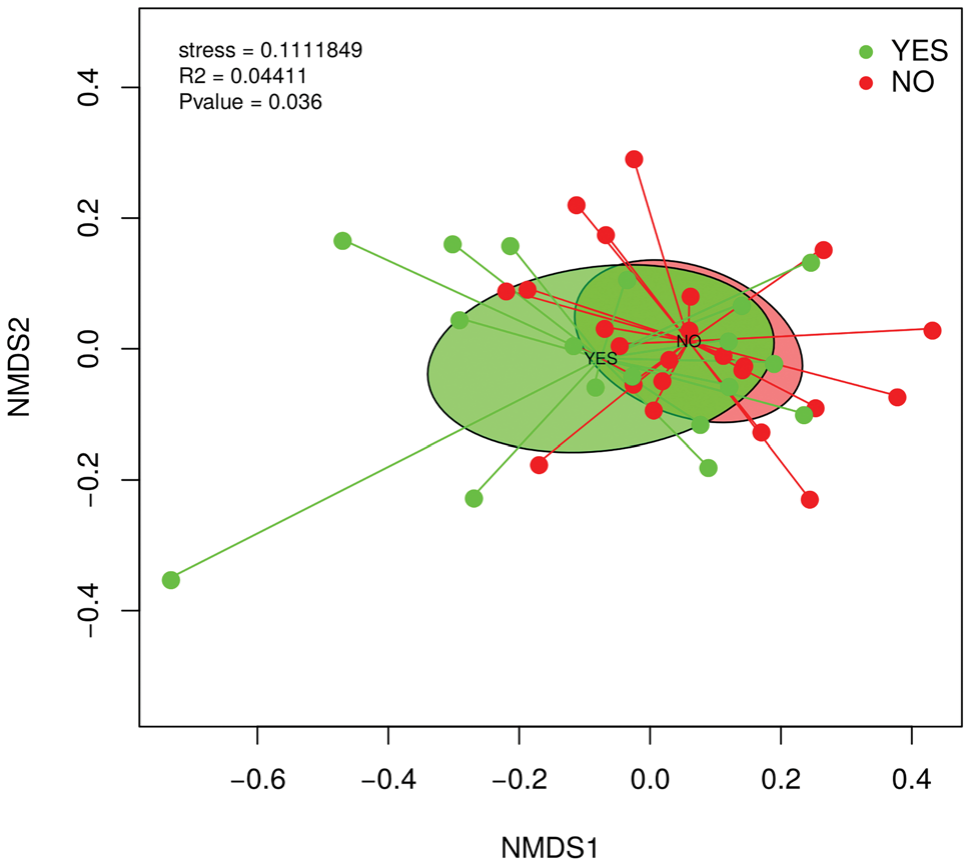
Comparison of contigs profile between the presence (yes) or absence (no) of PPSP.

The comparison of the contigs profile of the samples by using the Bray–Curtis dissimilarity index revealed the combined influence of sample type with respect to PPSP group (adonis2, *p* < 0.036) in the clustering and ordination of samples. The PPSP groups (“yes” and “no”) separated very well, and they are also statistically significant, but this variation explains 4.41% (Fig. 9).

### LEfSe

The same statistical analysis was performed with the contigs as previously with the raw data with the aim to establish the statistically significant relevant species in three groups using the SF-MPQ_C1 questionnaire; 60 min postoperatively, 12 weeks postoperatively, and the presence or absence of PPSP. The SF-MPQ_C1 questionnaire was characterised by 4 different levels: no pain, mild pain, moderate pain, and severe pain. Statistical differences are made by means of these 4 grades, but also with respect to the simple scale of the presence or absence of pain.

**Figure 10:**
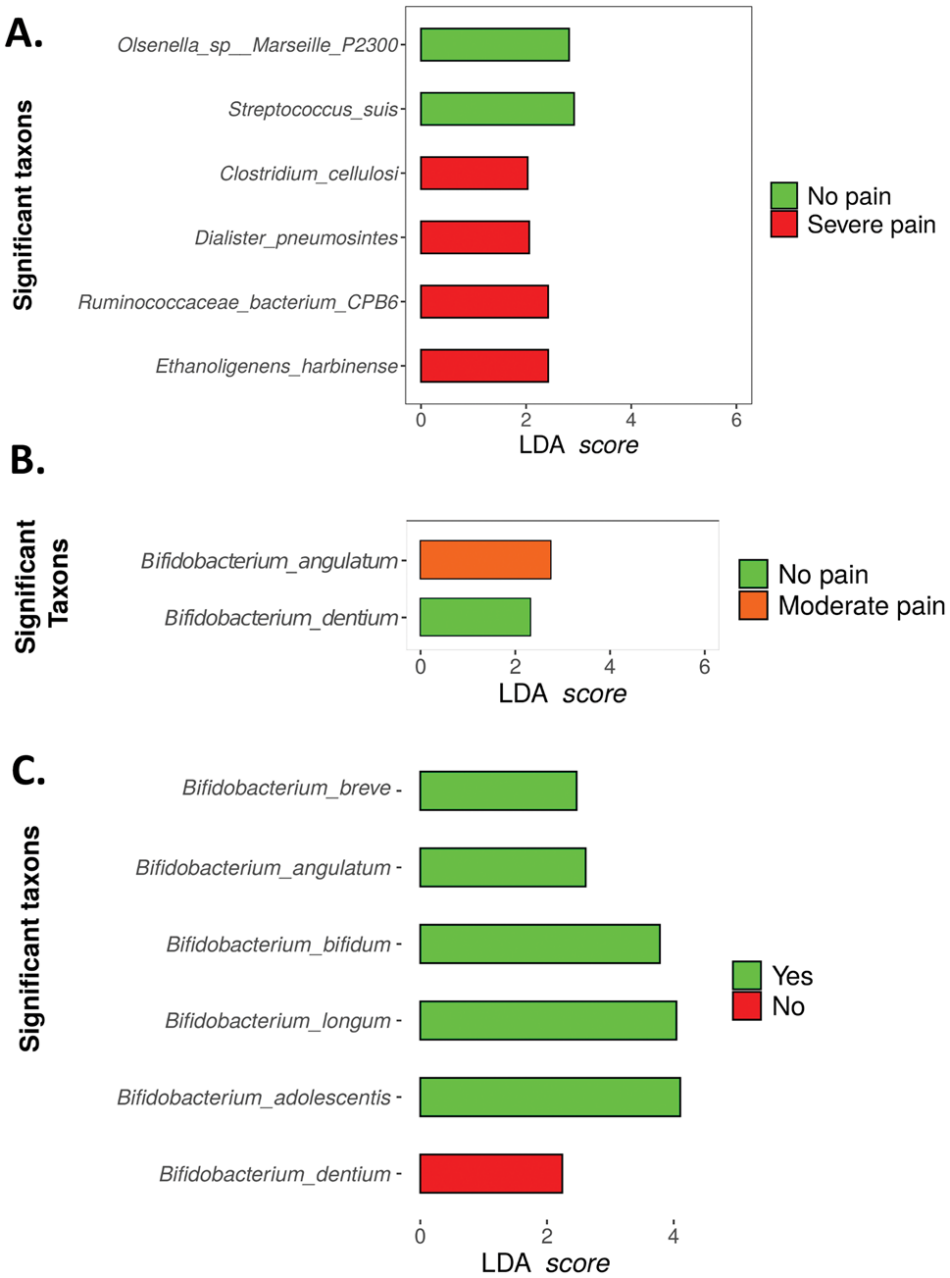
Lefse analysis revealing differential abundance of taxa between (**A**) Severe versus no pain group 60 min postoperatively (**B**) Moderate versus no pain group 12-weeks postoperatively (**C**) Presence (yes) or absence (no) of PPSP.

In the SF-MPQ_C1 questionnaire 60 min postoperatively, significance was noted in several species including *Ethanoligenens harbinense*, *Ruminococcaceae bacterium CPB6*, *Dialister pneumonintes,* and *Clostridium cellulosi* in the group of “severe pain”, whereas in the group “no pain”, *Streptococcus suis*, and *Olsenella marseille P2300* were found to be significantly related to this group (Fig. 10A). No further significant species were found in the rest of the groups. After 12 weeks postoperatively, the SF-MPQ_C1 questionnaire revealed significant results in the groups “moderate pain” (*Bifidobacterium angualtum*) and “no pain” (*Bifidobacterium dentium*), but no significantly different taxa were found in the rest of the groups (Fig. 10B). Finally, in the PPSP group there are two groups; presence (yes) or absence (no). Analysis revealed 6 significant species, one associated with the “no” group such as *Bifidobacterium dentium,* and another five species associated with the “yes” group such as *Bifidobacterium adolescentis, Bifidobacterium longum*, *Bifidobacterium bifidum*, *Bifidobacterium angulatum,* and *Bifidobacterium breve* (Fig. 10C).

### Spearman correlation between gut microbiota and PPSP in contigs

**Figure 11:**
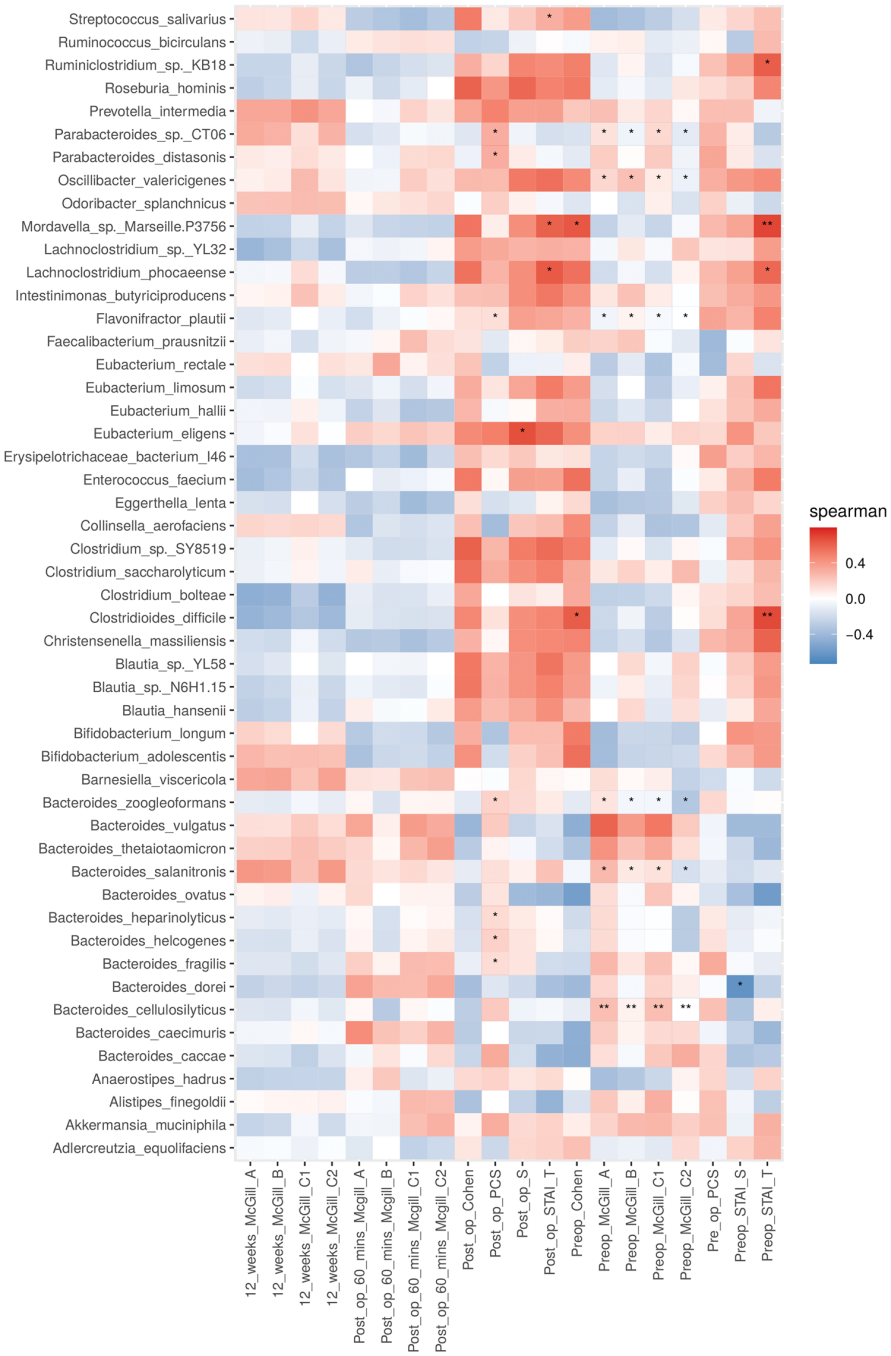
Spearman correlation between gut microbiota and PPSP in contigs. *p ≤ 0.05, **p ≤ 0.01.

The heatmap (Fig. 11) represents the contigs using a real-valued similarity matrix showing specific associations between the microbes and all pain indices examined in this study for each participant. A number of significant correlations were found between the indices pre-operative McGill C2, pre-operative McGill C1, pre-operative McGill B and pre-operative McGill A and the species *Bacteroides cellulosilyticus*, *Bacteroides salanitronis*, *Bacteroides zoogleoformans*, *Flavonifractor plautii*, *Parabacteroides sp. CT06* and *Oscillibacter valericigenes*. Depending on the index or species, there are positive or negative correlations, but all these species are repeated in these indices. A species to consider is *Clostridioides difficile*, which results in two significant positive correlations in the pre-operative STAI T and pre-operative Cohen indices. However, other positive correlations were also found, such as *Ruminiclostridium *sp.* KB18* and *Lachnoclostridium phocaeense* with the pre-operative STAI T index, as well as for the post-operative STAI T index with the species *Lachnoclostridium phocaeense*. Positive correlations were also been found with the species *Bacteroides zoogleoformans*, *Eubacterium eligens* and *Flavonifractor plautii* regarding the post-operative STAI S index. Finally, positive correlations were found in the species *Parabacteroides sp. CT06* and *Parabacteroides distasonis* with respect to the post-operative PCS index.

### Distribution of species grouped by presence or absence of PPSP

**Figure 12:**
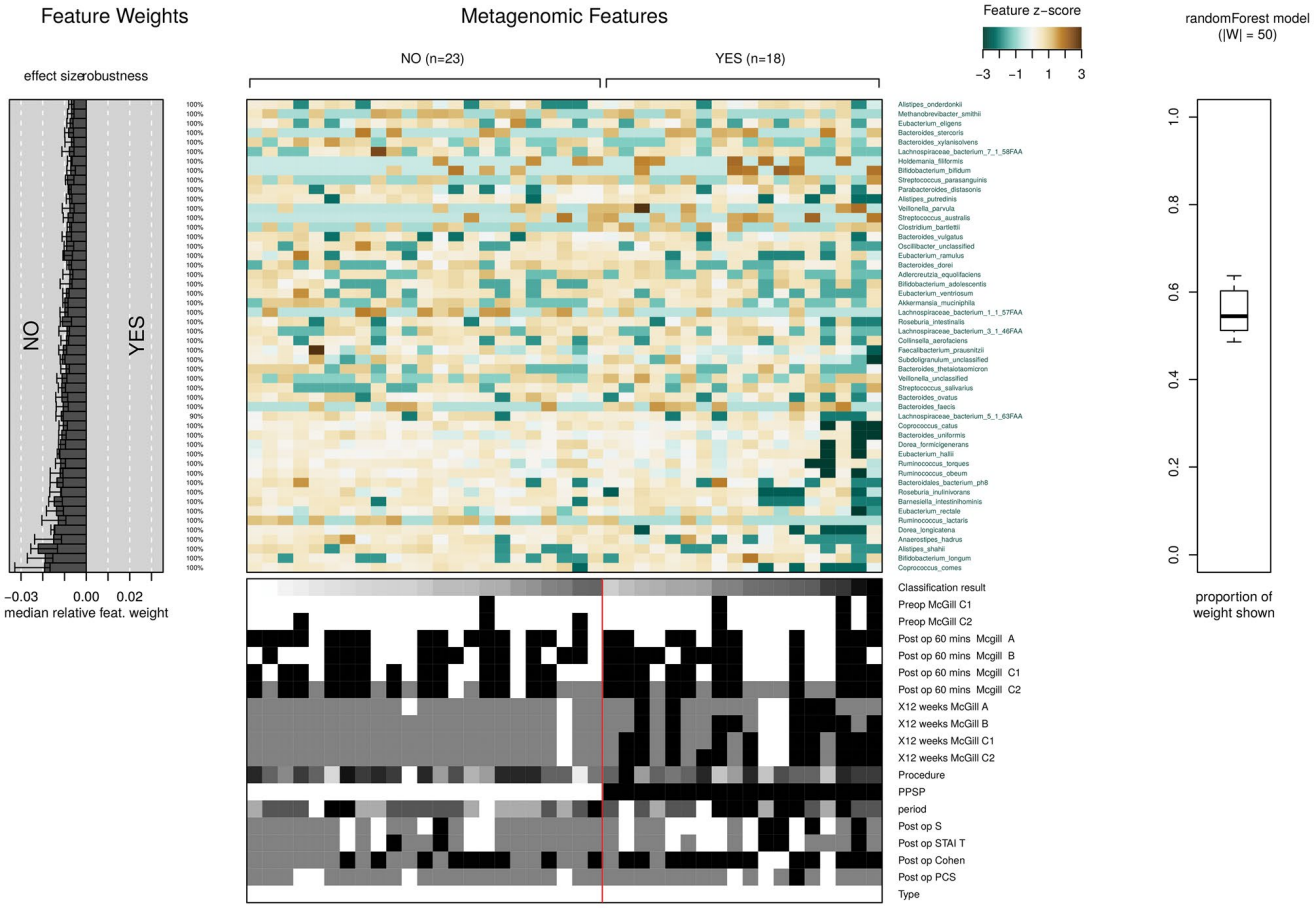
Random Forest demonstrating different distributions of species between the presence (yes) or absence (no) of PPSP groups.

The classifications of the groups presence (yes) or absence (no) of PPSP have been trained by means of Random Forest (RF) to distinguish between them (Fig. 12). This results in a depiction showing different distributions of species belonging to these groups, as well as different median relative feature weight for the species and the proportion of the weight of this model for the species with respect to the model, being around 50% and below the distribution of metadata among the samples. The final plot produced is the model interpretation plot. The plot shows for the top selected features the Z-Score. Very high (or very low) Z-scores are found in the tails of the normal distribution, and the further a data point is from the mean, the lower its probability of occurrence. This is what we see in this graph, totally different Z-scores between the PPSP groups, so being far from each other, there is a greater probability of significance between species from the two different PPSP groups.

## Discussion

Previous work has linked the diversity and composition of the gut microbiota with post-operative pain following upper limb surgery^35^. Here, for the first time we identify several species of bacteria that are associated with the presence or absence of PPSP following surgery for breast cancer. Our findings relate to early acute, and persistent (12 weeks) post-operative pain. These findings are of importance for the potential management of post-operative pain in a clinical setting.

The gut microbiota is known to play a major role in the perception of other forms of pain. For instance, the gut microbiota has been shown to modulate the visceral pain response in male mice^59^. Interestingly, and of relevance to the female-predominance of disorders of pain, the gut microbiota has been shown to be necessary for the oestrous cycle stage-dependent changes in visceral sensitivity^60^. Moreover, clinical visceral pain syndromes (such as IBS) are frequently characterised by a decreased abundance of Lactobacillus and Bifidobacterium^61^ as well as alterations in the Firmicutes:Bacteroidetes ratio^62^. Faecal microbiota transplantation in animal studies suggests that a healthy and diverse gut microbiota is important for pain regulation^63^. A growing evidence base also exists which describes the relationship between gut microbiota composition and other (non-visceral) forms of chronic pain such as neuropathic and inflammatory pain, and headache^32^.

Microbiota-derived mediators can enhance the development of chronic pain through peripheral sensitisation either directly (e.g. *Lactobacillus reuteri* DSM 17938 decreases jejunal spinal nerve firing via TRPV1 channel activation in mice stimulated using capsaicin or distension^64^) or indirectly by causing host immune cells to release pro-inflammatory cytokines or chemokines^65^. Pathogen-associated molecular patterns derived from the gut microbiota are key effectors of both direct and indirect forms of peripheral sensitisation^32^. This evidence further supports a role for the gut microbiota in pain processing and perception.

At present, the mechanisms behind the development of PPSP are incompletely understood, as is the role of the gut microbiota. Recently, it has been proposed that an imbalance in the gut microbiota, and therefore in production of microbial metabolites responsible for homeostasis, may play a role in the development of PPSP and influence the response to surgery or interventions^30^. To address this gap in knowledge, this study investigated any relationships between gut microbiota composition and PPSP. Further, the reported prevalence of PPSP after breast cancer surgery varies substantially; moderate quality evidence exists to suggest that it is greater following axillary lymph node dissection (versus sentinel lymph node biopsy) and after surgery that includes reconstruction^66–68^. For that reason, axillary lymph node dissection and procedures including reconstruction were not included in this study.

Analysis of the gut microbiota revealed a significant decrease in 3 alpha diversity measures in those patients reporting severe pain at the 60 min and the 12-weeks timepoints. A cluster of taxa represented by *Bifidobacterium longum* and *Faecalibacterium prausnitzii* was closely associated with those individuals reporting no pain at 12 weeks post-operation while *Megamonas hypermegale*, *Bacteroides pectinophilus*, *Ruminococcus bromii,* and *Roseburia hominis* clustered relatively closely in the group of patients fulfilling the criteria for persistent post-operative pain. A previous study has reported a lower relative abundance of *Faecalibacterium prausnitzii* in patients with fibromyalgia^69^. In agreement with our results, *Bifidobacterium longum* administration has been shown to reduce pain and inhibit pain behaviour in a rat model of osteroarthritis^70^. Contrary to our findings, *Roseburia hominis* has been previously reported to reduce visceral hypersensivity in rats where it was associated with the presence of PPSP in our study^71^. However, this reduction in visceral hypersensitivity has been suggested to be due to the normalisation of the gut microbiota community which may be disturbed in cases of pain^72^. Future studies are needed to fully interrogate the role of specific bacterial species in mechanisms of pain.

## Conclusion

We report here for the first time that shifts in the gut microbiota are associated with PPSP. Moreover, we highlight the robust differences in gut microbiota composition between those individuals with and without this PPSP. We interpret the partially positive results that we report here as indicative that an associative signal is detectable despite the effects of unmeasured confounding. We conclude that these results justify more detailed studies of specific clinical settings and also provide indicators of which (composition or abundance) gut microbiota features should be scrutinised. Such investigations could shed light not only on the magnitude of observed associations, but also on potential mechanisms underlying them. These findings highlight the role of the gut microbiota in PPSP.

## Limitations

While this study provides valuable insights into various microbial communities and taxa that relate to the presence or absence of PPSP, no causative links between these microbes and PPSP can be drawn. As the sample size of this study was modest, the results presented here would benefit from further verification in future studies. Further, while a previous study has shown differences in the prevalence of PPSP dependent upon the type of breast cancer surgery, we did not assess these differences. We and others have categorised several confounding factors as clinical (including demographics, surgical procedure type, and disease features), neurophysiological/pain, psychological, and genetic. For the setting of this study (women undergoing breast cancer surgery), the number of potential confounders is great (certainly in excess of one hundred). As this is one of the first examinations of the relationship between somatic pain and gut microbiota composition, our intention was to determine if this previously unconsidered factor might influence the development of PPSP. Future studies should assess the efficacy of microbiota-targeted therapies in the reduction of PPSP.

### Supplementary Information

Supplementary Figure S1.

## Data Availability

The data generated and/or analysed during the current study are available from corresponding author on reasonable request.

## Abbreviations

ASA: American Society of Anaesthesiologists
BMI: Body mass index
CNS: Central nervous system
EAMMS: End anchored max scoring segments
FDR: False discovery rate
IBS: Irritable bowel syndrome
IV: Intravenous
LEfSe: Linear discriminant analysis effect size
LFC: Log2 fold change
MDS: Multidimensional scaling analysis
PCS: Pain catastrophising scale
PERMANOVA: Permutational analysis of variance
PPI: Present pain intensity
PPSP: Persistent post-surgical pain
RF: Random forest
SF-MPQ: Short-form McGill questionnaire
SNLB: Sentinel lymph node biopsy
STAI: State-trait anxiety inventory
TLR: Toll-like receptor
WLE: Wide local excision
