# Supplementary material for: The gut microbiota in persistent post-operative pain following breast cancer surgery
Source: Sci Rep. 2024 May 30;14:12401. doi: 10.1038/s41598-024-62397-1 (PMC11137075; doi:10.1038/s41598-024-62397-1)
Supplement: Supplementary file 1 — Supplementary Figure S1. [file 41598_2024_62397_MOESM1_ESM.pdf]

Supplementary material

StrainPhlAn

The most interesting species related to pain identified in this study were *Bifidobacterium adolescentis*, *Bifidobacterium longum*, *Escherichia coli,* and *Faecalibacterium prausnitzii* (identified in most of the samples belonging to this study at a sufficient coverage to be profiled by StrainPhlAn). The strains were profiled according to the PPSP group or the reference strains. To further determine if the taxonomic differences at the species level between these groups extended to the strain level, further analysis of the assigned reads of *Bifidobacterium adolescentis*, *Bifidobacterium longum*, *Escherichia coli,* and *Faecalibacterium prausnitzii* were carried out. This revealed that the strains were also grouped phylogenetically according to the different groups (Figure S1 A-D).

The StrainPhlAn inferred phylogeny highlighted the presence of three clusters of *Bifidobacterium adolescentis* strains with respect to PPSP (presence (yes) or absence (no)) they occupy different strains in the tree, are heterogeneous and able to have completely different functions that are involved in the pain pathway. Three clusters of *Faecalibacterium prausnitzii* were identified. *Bifidobacterium longum* is divided into 10 clusters, most of them occupied by the reference genomes taken, only 4 clusters are populated by samples, more presence for pain samples than for those without, occupying different clusters. Finally, only three samples clustered for *Escherichia coli*, perhaps more depth of sequencing would have been needed to find more, however it shows how different between groups they are in the phylogenetic tree, showing how heterogeneous and different they are.

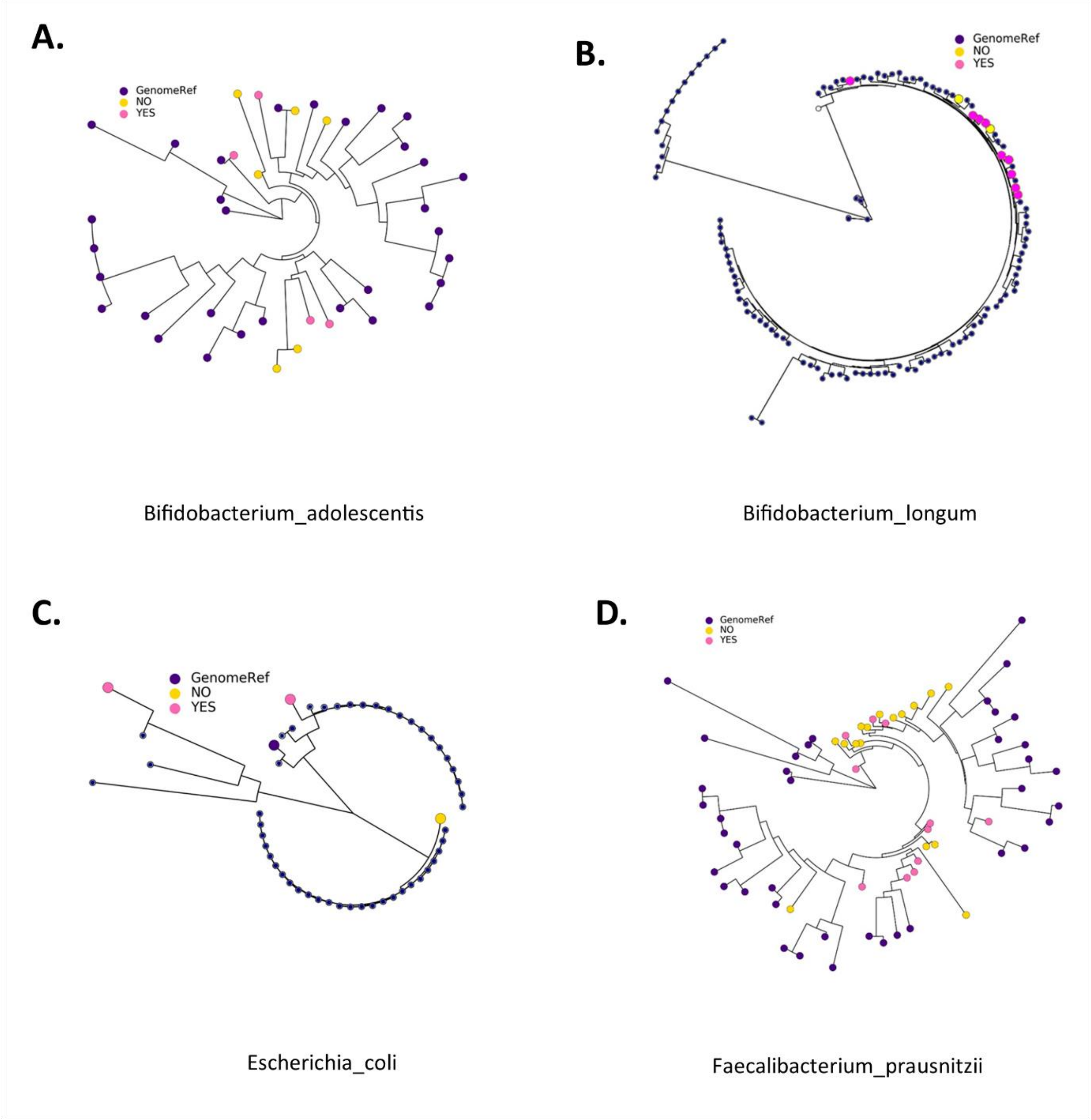

Figure S1: StrainPhlAn inferred phylogeny for (A) Bifidobacterium adolescentis (B) Bifidobacterium longum (C) Escherichia coli (D) Faecalibacterium prausnitzii.
